# Prevalence and Antibiogram Assessment of* Staphylococcus aureus* in Beef at Municipal Abattoir and Butcher Shops in Addis Ababa, Ethiopia

**DOI:** 10.1155/2018/5017685

**Published:** 2018-05-06

**Authors:** Feben Adugna, Mahendra Pal, Gebrerufael Girmay

**Affiliations:** ^1^Addis Ababa University College of Veterinary Medicine and Agriculture, Addis Ababa, Ethiopia; ^2^Ethiopian Institute of Agricultural Research, Addis Ababa, Ethiopia

## Abstract

**Objective:**

A cross-sectional study was conducted from October 2013 to April 2014 to determine the prevalence and antibiotic resistance of* Staphylococcus aureus* from beef of Addis Ababa Abattoir and butcher shops in Addis Ababa. Seven hundred sixty-eight swab samples were taken from the abattoir and butcher carcasses using a systematic random sampling. One hundred twenty swab samples were also taken from hooks, cutting tables, and knives from the abattoir.* Staphylococcus aureus* positive isolates were taken for antibiotic susceptibility test. A questionnaire survey was conducted in the abattoir and butcher workers to assess the hygienic practice and possible risk factors regarding the contamination of meat.

**Results:**

The prevalence of* S. aureus* in the abattoir, butcher, cutting table, hook, and knife was 9.4%, 19.8%, 15%, 15%, and 22.5%, respectively. The prevalence of* S. aureus* in the knife and butcher was found to be 2.8 (OR = 2.8, CI = 1.2–6.4) and 2.4 (OR = 2.4, CI = 1.6–3.6) times that of the abattoir results (*p* < 0.01). The antimicrobial susceptibility testing was also conducted on 133 isolates of* S. aureus* using the disc diffusion susceptibility method. Bacitracin, neomycin, and methicillin were found to be 100% resistant to* S. aureus*. To avoid the presence of pathogenic* Staphylococcus *isolates, preventive measures using good hygienic practices during slaughtering and handling of the beef carcasses are recommended.

## 1. Introduction

Ethiopia was assumed to have about 59.5 million cattle population [1]. These cattle produce more than 3.6 million tonnes of milk and about one million tonnes of meat annually [2]. Foodborne diseases occur in developing countries because of the poor food handling and sanitation problems [3]. Although animal's tissue is sterile, during slaughtering, microorganisms could contaminate the tissue primarily from the exterior or the interior environments [4, 5].

*Staphylococcus aureus *is one of the food borne diseases transmitted from the contaminated animal source food staffs [6]. It produces heat stable and proteolytic enzyme resistant enterotoxins that cause food poisoning in humans leading to vomiting, abdominal pain and diarrhea [7].* Staphylococcus aureus* is found in 30% nonclinical nasal carrier population [8]. This could be the sole source of contamination in abattoir and butcher workers for those who do not have enough awareness on the nature of the disease.

Ethiopian raw beef consumption habit is the potential cause of foodborne illnesses [5, 9]. Raw meat is available in open-air local butchers without the cold-chain process and purchased by consumers. Meat processing at butchers is likely to contribute for the contamination of minced beef meat as compared to the carcasses [10].

Although it is difficult to prove the role of drug resistance in bacterial contaminating food with increased clinical cases, the presence of such bacteria in food items could play a great role in the spread of antimicrobial resistance. Thus, adequate information should be gathered to develop an effective strategy to reduce the foodborne illness and drug resistance [11].

The objective of this study was to determine the prevalence of* S. aureus *in abattoir, butchers, and equipment. Antimicrobial resistance of* S. aureus* from the beef abattoir and butchers and awareness of the abattoir and butcher workers will also be assessed.

## 2. Main Text

### 2.1. Methods

#### 2.1.1. Study Area

The study was conducted in Addis Ababa city which has an average altitude of 2000–2560 masl. It has average of 1100 mm and the highest rain falls from June to September.

#### 2.1.2. Study Approach

A cross-sectional study was employed to determine the prevalence of* S. *a*ureus* and antibiotic susceptibility from beef meat at the butchers and Addis Ababa abattoir (November 2013 to April 2014). A prestructured questionnaire survey was conducted to assess the status of the food hygiene and sanitation in the abattoir and butchery.

The expected prevalence was assumed to be 50% as there were no previous studies. Ninety-five percent confidence level at 5% precision was employed to determine the sample size [12]. So, 384 for the abattoir, 384 for butchers, and 120 swabs from abattoir equipment were collected.

**Figure 1:**
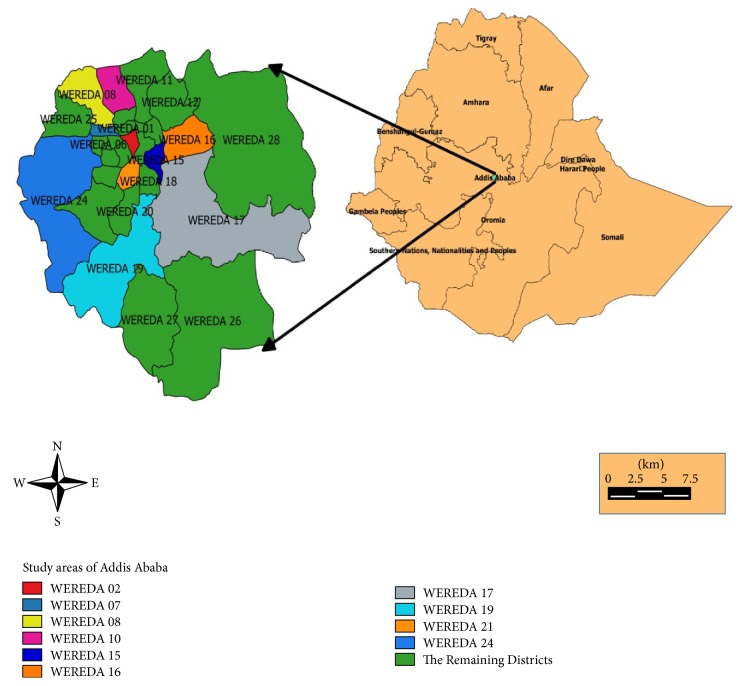
Map of Ethiopia, Addis Ababa, and study districts (weredas).

A systematic random sampling was employed to select swabs from the abattoir and butchers after lists of animals from the ante-mortem inspection and lists of butcher houses from the Addis Ababa abattoir were found. The lists of districts (weredas) where butcher samples were taken are provided in Figure 1 (given in EPS file). Convenience sampling was used to take swab samples from different types of equipment of the abattoir. Pure isolates of* S. aureus* from the positive samples were taken for antimicrobial resistance test.

A questionnaire survey was conducted on meat shop and Addis Ababa abattoir workers to assess the hygienic practices. Semistructured questions were prepared and pretested on 5 people. Questions were originally written in English and translated into the Amharic language when administered.

#### 2.1.3. Sample Collection and Processing

Samples were collected from the butchers and abattoir and swabbed using the method described in ISO6888-2 placing the sterile template on specific sites of a carcass. Sterile cotton tipped swab fitted with shaft was soaked in buffered peptone water (Oxoid Ltd., Hampshire, England) and rubbed horizontally and vertically on the carcasses. Abdomen, thorax, crutch, and breast sites which have the highest contamination (ISO6888-2) were chosen for sampling [13]. After rubbing completed, the shaft was broken against the inner wall and disposed to leave the cotton swab in a test tube.

#### 2.1.4. Isolation and Identification

Staphylococci were isolated and identified through the primary (culture, gram staining, catalase test, oxidase test, and oxidation-fermentation) and secondary identifications (coagulase, mannitol salt agar, purple agar base, and DNase agar tests) according to the standard techniques [13–15].

#### 2.1.5. Antimicrobial Susceptibility

Isolates were tested for 13 commonly used antimicrobials for the susceptibility tests using Kirby-Bauer disk diffusion method using 0.5 McFarland standards on Muller Hinton agar plats [16].

Colonies isolated from pure culture were transferred to 5 ml tryptone soya broth. Turbidity of the broth was adjusted by adding sterile saline to obtain a turbidity visually comparable with 0.5 McFarland standards. The Muller-Hinton Agar (MHA) plates were prepared using sterile cotton swabs dipped into the tryptone soya broth culture and then the surfaces of MHA plate were swabbed.

Antibiotic discs, amoxicillin (10 *μ*g), bacitracin (10 *μ*g), cephalothin (30 *μ*g), chloramphenicol (30 *μ*g), clindamycin (30 *μ*g), cloxacillin (12.5 *μ*g), erythromycin (15 *μ*g), methicillin (5 *μ*g), neomycin (30 *μ*g), nitrofurantoin (15 *μ*g), norfloxacin (10 *μ*g), penicillin G (10 units), polymyxin B (10 *μ*g), rifampicin (5 *μ*g), and vancomycin (30 *μ*g), were placed on the agar plate using sterile forceps and pressed gently to ensure complete contact with the agar surface. These antibiotic discs were purchased from Oxoid, England. The plates were incubated for 24 hours at 37°C aerobically. Inhibition zones were measured and interpreted as susceptible, intermediate, and resistant according to NCCLS [17].

The inhibition zones were reported as the diameter of the zone of surrounding the individual disk in which bacterial growth was absent. The isolates were defined as resistant, intermediate, and susceptible according to the manufacturer's manual [17].

A questionnaire survey was conducted on beef meat shop and Addis Ababa abattoir workers to assess the hygienic practices. Semistructured questions were prepared and pretested on 5 people. Questions were originally written in English and translated into the Amharic language when administered.

#### 2.1.6. Data Analysis

Data were entered into excel sheet, organized, and analyzed using STATA/IC 13.1. The overall prevalence of* S. aureus* in beef meat carcasses, butcher shops, and equipment was determined using logistic regression. The odds ratio was used to indicate the strength of association. *p* value < 0.05 was considered as statistically significant.

## 3. Results

### 3.1. *Staphylococcus aureus* Prevalence

The prevalence varied between sample sources and among sample types. The highest was recorded from the knife and followed by the butcher shops (Table 1).

A knife was found to have the highest prevalence (22.5%) followed by the butcher shops (19.8%) (Table 1). The least prevalence of* S. aureus* was found in the abattoir (9.4%) comparing with the butcher shops and knife prevalence. Prevalence in the butcher shops was higher than the abattoir by 2.4.

### 3.2. Antimicrobial Susceptibility

One hundred and thirty-three* S. aureus *isolates were tested to various antimicrobials using the disc diffusion technique. The resistant pattern varied among the thirteen drugs. The isolates were completely susceptible to the chloramphenicol, clindamycin, and ampicillin. On the contrary, all isolated strains were found to be resistant to bacitracin, neomycin, and methicillin and 95% of the isolates to tetracycline. It was noticed that 49.5%, 45.5%, 45%, and 13% of the strains were also resistant to penicillin G, vancomycin, cloxacillin, and norfloxacin, respectively, while 86.5%, 73%, 72%, 54%, and 50% of the strains were susceptible to amoxicillin, norfloxacin, erythromycin, cloxacillin, and penicillin G, respectively. Intermediate susceptibility was observed in vancomycin (54%) and erythromycin (27%). Amoxicillin and norfloxacin showed equal intermediate susceptibility (13%) and small intermediate susceptibility was demonstrated in tetracycline (Table 2).

### 3.3. Hygienic Practice of Butcher Shop Workers

About 24 butcher shop workers were interviewed to assess their hygienic practice. Among them, 58.3% were literate and 41.7% had not been trained for butcher hygiene. The study showed that 75% of the workers at the butcher shops did not wear aprons and 58.3% of them did not cover their hair; 65% of the butcher shop owners did not have cashier and serving food. It was observed that 41.1% of the butcher shop workers used only water for cleaning (Table 3).

### 3.4. Knowledge of Abattoir Workers on the Hygienic Practices

Out of the 24 abattoir workers, 58.3% of them were not educated; however, all of them get training regarding meat and personal hygiene. The study showed that 83.4% of the abattoir workers used aprons and 91.7% of them were used to cover their hair. However, 83.3% of the abattoir workers' protective cloths, which have direct contact with the meat, were dirty. It was also noticed that 100% of the workers used water and soap for cleaning purpose. Furthermore, only 33.3% of the workers remembered to disinfect their knives between consecutive works. It was also observed that 58.3% of the workers were doing their work having minor skin wounds.

## 4. Discussion

Similar findings with our result were reported from Ethiopia and Nigeria [10, 18]. This could be because of the similarity of the study with our result as both of them work on meat and food handlers. Moreover, de Boer et al. stated comparable results from the abattoir and butcher shops with similar approach of ours [19]. On the contrary, lower prevalence of* S. aureus* (1.3%) was reported from Nigerian abattoir conducted by Iroha and his coworkers [20]. This could be due to the time of collecting the samples in that they conducted their work at the festive times and samples were collected within 8 hours after slaughter and during early in the afternoon in order to minimize contamination and postslaughter timings.

Goja and his coinvestigators isolated* S. aureus* from beef meat in Sudan and also found a lower prevalence (12%) than ours [4]. This could be due to the fact that they collected the sample as fresh and immediately processed in the laboratory as they isolated only forty samples. On the other hand, Gurmu and Gebretinsae isolated from butcher shops in Ethiopia and found higher prevalence than our finding (28%) [21]. The type of samples taken (hands, tables, and knives) and the relatively lower cleaning exercise could be attributed to the higher prevalence in their areas. In this study,* S. aureus* was isolated in butcher shops (19.7% and 17.6%) equipment which is similar to Bhargava et al. [22]. Ahmad and coworker of Egypt, isolated higher prevalence in a beef outlet (70%) than beef abattoirs (55%) [23]. This accords with our result in that higher prevalence of the disease is observed in the butcher shops than the abattoirs because of the continuous contamination through the transportation process.

Prevalence of antimicrobial resistance increased during the recent decades [24, 25]. Bacitracin, neomycin, and methicillin were identified as totally ineffective for* S. aureus* bactericidal drugs. Our finding is comparable with Iroha and his coworkers that* S. aureus was* susceptible to clindamycin and ampicillin and had lower susceptibility to erythromycin and amoxicillin [20]. Çepoğlu and his coworkers discovered that 4.7% of* S. aureus *isolates were resistant to methicillin, 1.2% to vancomycin, 33.3% to erythromycin, and 29.1% to tetracycline and 3.5% isolates showed intermediate resistance to methicillin and 2.4% to vancomycin [26].

Adesiji et al. reported that isolates of* S. aureus *were susceptible to erythromycin and vancomycin, which is inconsistent with our study in which 72% of the isolates were susceptible to erythromycin and 54% of the isolates were intermediately susceptible to vancomycin [27]. The current data, similar to Barena and Fetene, demonstrated beef meat and equipment were frequently contaminated with multidrug-resistant* S. aureus* [28].

Ninety percent of the* S. aureus* isolates from Ethiopia were found to be methicillin resistant. This finding was consistent with the present study, in which 100% methicillin resistance was recorded in all isolates [10]. In our study, the resistance rate of* S. aureus* to tetracycline was higher than the findings reported in Ethiopia [29]. In addition, lower degree of resistance to tetracycline was observed in Italy (58%), North Palestine (45%), South India (11.8%), and USA (23%) [30–33].

Foodborne diseases occur in developing countries because of the poor food handling and sanitation practices [34]. Animal food products are regarded as a high-risk commodity with respect to pathogens and other contaminants [35]. Hygienic practices and quality control methods of meat and meat products are recommended in many countries [36, 37].

From the butcher shops, 41.7% of the respondents were illiterate and 58.3% of the respondents did not take the training on butcher shops and personal hygiene. About 58.5% of the workers did not use hair cover; at the same time, 75% were not wearing an apron and 65% butcher shops did not have cashier which only focused on the management of their hands and the equipment.

Slightly similar results were reported in Mekelle that 48% of the respondents did not have a cashier; 78% of the respondents did not take training courses regarding meat and butcher hygiene. Educational status is almost similar to the present finding in which 58% of the workers were illiterate [3].

Another study from Mekelle by Gurmu and Gebretinsae demonstrated that 41.7% were illiterate and 58.3% of them did not take training courses [21]. Another study also showed that 41.7% of the butcher workers did not wear aprons and 58.3% did not cover their hair [38].

About 75% of butchers did not wear aprons and 58% did not cover their hair. The findings disagree with reports from South Africa (85%) [39]. It is also indicated that 25% of the butchers handled money while serving food. Muinde and coworkers from Kenya also showed 91.7% of butchers handled money while serving food that could be the possible source of* S. aureus *contamination [40].

In conclusion, the present study confirmed that there is significantly higher* S. aureus *contamination of beef meat while transferring from the abattoir (9.4%) to the butcher houses (19.8%). The highest source of contamination could be the abattoir workers as knives caught by the hands of these workers were contaminated even beyond (22.5%) the* S. aureus* prevalence in the butcher houses. As human nose is the main colonization site of* S. aureus, *approximately 30% of workers noses are colonized, and chronic nasal carriages even worsen the risk of infection by* S. aureus *[8]. In addition, the lower educational level of the abattoir workers and the limited trainings given to the butcher workers on the subject matter could contribute for the higher* S. aureus* contamination of butchers' beef meat in the Addis Ababa city. On the other hand, antimicrobial resistance is becoming the headache of the world. Our result has confirmed that 100% resistance of the three commonly used drugs means that we should give due emphasis to solve the sole problem.

## 5. Limitation

Backyard slaughtering is common in Ethiopia, which can affect the result comparing the prevalence of* S. aureus* in abattoir and butcher shops as sources of butcher shops could be from backyard slaughtering. Similarly, the source of drug resistance is difficult to determine as there is a lack of awareness of the appropriate usage of antibiotics. Considerable patients and animal owners discontinue finishing the prescribed antibiotics, which leads to the development of resistance.

## Figures and Tables

**Table 1:** The overall prevalence of *Staphylococcus aureus* from different materials.

Sample type	Total samples	Positives	Prevalence (%)	95% CI For prevalence	OR	95% CI for OR
Abattoir	384	36	9.4^a^	5.8–12.9	1	
Butcher	384	76	19.8^b^	16.2–23.3	2.4	1.6–3.6
Cutting table	40	6	15^ab^	4.0–26	1.7	0.7–4.3
Hook	40	6	15^ab^	4.0–26	1.7	0.7–4.3
Knife	40	9	22.5^b^	11.5–33.5	2.8	1.2–6.4

*Total *	*888*	*133*	*15*			

*Note*. ^a, b, ab^prevalences with the similar letters are not statistically significant at 95% confidence level. CI = confidence interval; OR = odds ratio.

**Table 2:** Antimicrobial susceptibilities among the 133 isolates of *S. aureus*.

Antimicrobials	Susceptible	Intermediate	Resistant
	No (%)	No (%)	No (%)
Bacitracin	0	0	133 (100)
Neomycin	0	0	133 (100)
Methicillin	0	0	133 (100)
Tetracycline	0	6 (4.5)	127 (95.5)
Penicillin G	67 (50.5)	0	66 (49.5)
Vancomycin	0	72 (54.5)	61 (45.5)
Cloxacillin	73 (54.8)	0	60 (45.2)
Norfloxacin	97 (73)	18 (13.5)	18 (13.5)
Erythromycin	97 (72.9)	36 (27.1)	0
Amoxicillin	115 (86.5)	18 (13.5)	0
Chloramphenicol	133 (100)	0	0
Clindamycin	133 (100)	0	0
Ampicillin	133 (100)	0	0

**Table 3:** Knowledge and skill of butcher shop workers on hygienic practices.

Observation type	Values	Frequency	Percent (%)
Educational status	Grades 1–8	8	33.3
	Grades 8–10	6	25
	Illiterate	10	41.7

Training	Yes	14	58.3
	No	10	41.7

Money	Cashier money handler	6	35
	Butcher money handler	18	65

Cleaning	Water only	10	41.5
	Water and soap	14	58.5

Hair cover	Not covered	14	58.5
	Covered	10	41.5

Apron	Not used apron	18	75
	Used apron	6	25

## Data Availability

The datasets used during the current study are available from the corresponding author on reasonable request.
